# Accessibility and surface area drive desert toad presence at artificial water sites in the Great Basin and Mojave Deserts

**DOI:** 10.1371/journal.pone.0357569

**Published:** 2026-10-07

**Authors:** Leah N. Gordillo, Nathalie Larios-Chavez, Gisel Larios, Robert N. Knight, Randy T. Larsen, Brock R. McMillan, Lucas K. Hall

**Affiliations:** 1 Department of Biology, California State University Bakersfield, Bakersfield, California, United States of America; 2 Air Force Nuclear Weapons Center, Sentinel System Program Office, Hill Air Force Base, Utah, United States of America; 3 Department of Plant and Wildlife Sciences, Brigham Young University, Provo, Utah, United States of America; Griffith University, AUSTRALIA

## Abstract

Artificial Water Sites (AWS) are human-made or modified water sources constructed or maintained for livestock, select wildlife game species, or other management purposes in arid environments. However, non-target species such as desert toads have been documented utilizing AWS. Because the reproductive ecology of desert toads relies on water, understanding factors explaining their presence at AWS can inform conservation practices. To address this lack of information, we examined how AWS design, spatial isolation, local terrain, and surrounding vegetation influenced toad presence in the Great Basin and Mojave Deserts. From May to June 2011, we surveyed 68 AWS for species presence of toads and quantified AWS design features and environmental variables. We detected Great Basin spadefoot toads (*Spea intermontana*) at 61% of Great Basin AWS and red-spotted toads (*Anaxyrus punctatus*) at 38% of Mojave AWS. We used generalized mixed-effects models and AIC model selection to identify the most informative variables. Top performing predictors resulted in the final model ‘access height (lowest distance between water edge and ground surface) + surface area (surface area coverage of water)’, which accounted for 86% of model weight. The predictor ‘access height’ had a significant negative association with toad presence whereas ‘surface area’ had a significant positive association. Spatial isolation, terrain, and vegetation predictors were not found to influence toad occurrence. Our results indicate that AWS design, specifically lower access height and increased surface area, enhances desert toad use, potentially improving amphibian habitat suitability and persistence.

## Introduction

Arid environments often have limited availability of free-standing water (hereafter ‘free water’), increasing the overall value of this resource for water-dependent species. Some species of wildlife, such as amphibians, rely on free water for habitat during at least part of their life cycle [1]. However, increasing anthropogenic water use coupled with increasing aridity due to climate change reduces availability of free water in deserts [2–4], with consequences for dependent species [5] such as desert toads.

The ecology of desert toads is inextricably linked to free water due to their water economy and reproductive biology. Desert toads face high rates of evaporative water loss due to their semi-permeable skin and exposure to harsh desert climates, including high temperatures and low humidity [6,7]. Desert toads behaviorally reduce water loss by avoiding direct solar exposure, being nocturnal, and using moist soils for burrows [8], but cannot effectively separate from free water [9]. During prolonged dry periods, many desert amphibians estivate underground, entering a state of reduced metabolic activity that limits water loss until favorable conditions return [10–12]. Emergence and reproductive activity are often closely associated with rainfall events [13]. Many desert toads exhibit explosive breeding, in which adults congregate at water sources, mate, and oviposit over a relatively short period following rainfall. Females deposit eggs in water, and the resulting larvae must complete development and metamorphosis before temporary breeding pools dry. Failure to metamorphose before pool drying can result in complete reproductive failure [14]. Thus, the availability and persistence of free water are particularly important to desert amphibian reproduction and population persistence. As a result, the loss of surface water reduces breeding habitat and may contribute to the current decline in amphibian populations in deserts [15,16] and worldwide [17,18].

Although water is generally scarce in deserts, artificial water sites (AWS) are human-made or modified water sources constructed or maintained for livestock, select wildlife game species, or other management purposes [19,20]. In our study, AWS included wildlife water catchments, livestock drinking troughs, and water treatment ponds. Although other artificial water sources, such as farm dams and agricultural ponds, can provide important amphibian habitat [21], these water sources were not present in our study. Although AWS are generally intended for livestock or wildlife, many nontarget species (e.g., desert toads) have been documented at these features [22–26]. While desert toads have been observed to opportunistically use available water, the factors that potentially influence the use of AWS by desert toads are not well understood. Though the use of AWS as habitat enhancement for native species has been debated [20,27–29], we lack an understanding of the factors that contribute to toad presence. If AWS are to be used as a mitigation for declining amphibian habitat in arid regions, determining characteristics that align with species use should be explored [30].

Several characteristics of AWS likely influence desert toad presence, including construction design and accessibility, spatial isolation from other water sources, local terrain and soil conditions, and vegetation in or around the water source. Because AWS are generally designed and maintained for livestock or game species, variation in access height, edge configuration, and construction material often reflects engineering decisions and site-specific constraints rather than considerations for amphibian access. Construction features such as smooth, steep, or vertical surfaces may limit access to water [e.g., 22, 31]. Spatial isolation may also influence use because many desert toads have relatively limited dispersal distances and home ranges [9,32–34]. Local terrain, soil conditions, and vegetation may further affect movement, availability of refuge from harsh climate and predators, and foraging opportunities [35–38]. These characteristics are not necessarily independent and may interact to influence whether desert toads use a given AWS.

The objective of our study was to test hypotheses potentially explaining the presence of toads at AWS. To test the following hypotheses, we sampled 68 AWS in the Great Basin and Mojave Deserts.

**Hypothesis 1: Construction design of AWS affects toad presence**

*Prediction*: The likelihood of toad presence will increase with AWS that have design characteristics facilitating access to water (e.g., reduced height between the wetted edge of AWS and the ground, rough surfaces that improve climbing, etc.).

**Hypothesis 2: Spatial isolation characteristics of AWS affect toad presence**

*Prediction*: The likelihood of toad presence will increase as the isolation of AWS decreases.

**Hypothesis 3: Local terrain surrounding AWS affects toad presence**

*Prediction*: The likelihood of toad presence will increase at AWS where the surrounding terrain (i.e., slope, terrain ruggedness, and topographic position derived from a 30-m digital elevation model within 90 m of each AWS) provides suitable burrowing substrates for aestivation and complex topographic features where rocky terrain may provide refugia.

**Hypothesis 4: Vegetation cover in and around AWS affects toad presence**

*Prediction*: The likelihood of toad presence will increase as vegetation cover increases in or around AWS as some toads use vegetation cover for refuge or foraging.

## Methods

### Study regions

The Great Basin study region included 915 km^2^ of land not publicly accessible and managed by the United States Department of Defense at the United States Army Dugway Proving Ground in west-central Utah (Fig 1). The terrain in this region was typical of Lake Bonneville lakebed, characterized by an expansive valley bottom with dune systems, alkaline flats dominated by black greasewood (*Sarcobatus vermiculatus*), and surrounded by north south oriented basin and range topography. Closer to the mountains, cheatgrass (*Bromus tectorum*) was common within communities of big sagebrush (*Artemisia tridentata*), rabbitbrush (*Chrysothamnus* spp.), and juniper (*Juniperus osteosperma*). Elevations ranged from 1320 m to 1760 m. Based on [1981–2010] PRISM climate normals spatially averaged across the study region, the Great Basin had a mean annual air temperature of 9.7°C. The annual averages of daily minimum and maximum temperatures were 2.4°C and 16.9°C, respectively, and mean annual precipitation was 364.6 mm [39]. We identified 18 AWS, including wildlife water catchments, water treatment ponds, livestock drinking troughs, within the Great Basin study region using locations provided by the United States Department of Defense and Utah Department of Natural Resources (Figs 1, 2).

**Fig 1:**
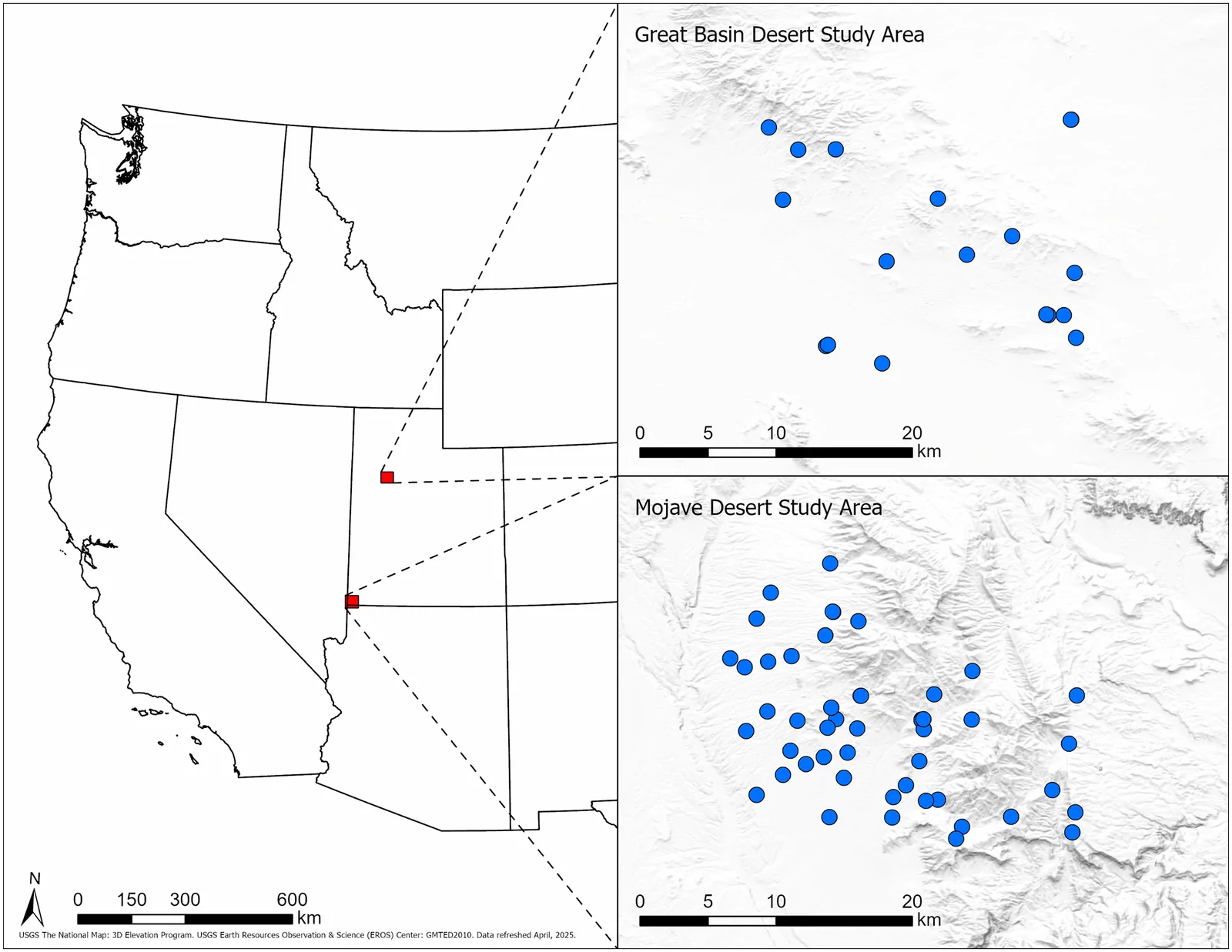
Location of study regions (red squares) with artificial water sites (blue circles) surveyed for Great Basin spadefoot toads (*Spea intermontana*) and red-spotted toads (*Anaxyrus punctatus*). Data were collected in the Great Basin and Mojave Deserts from 2011. Map created in ArcGIS Pro using USGS The National Map public-domain geographic data and USGS Shaded Relief.

**Fig 2:**
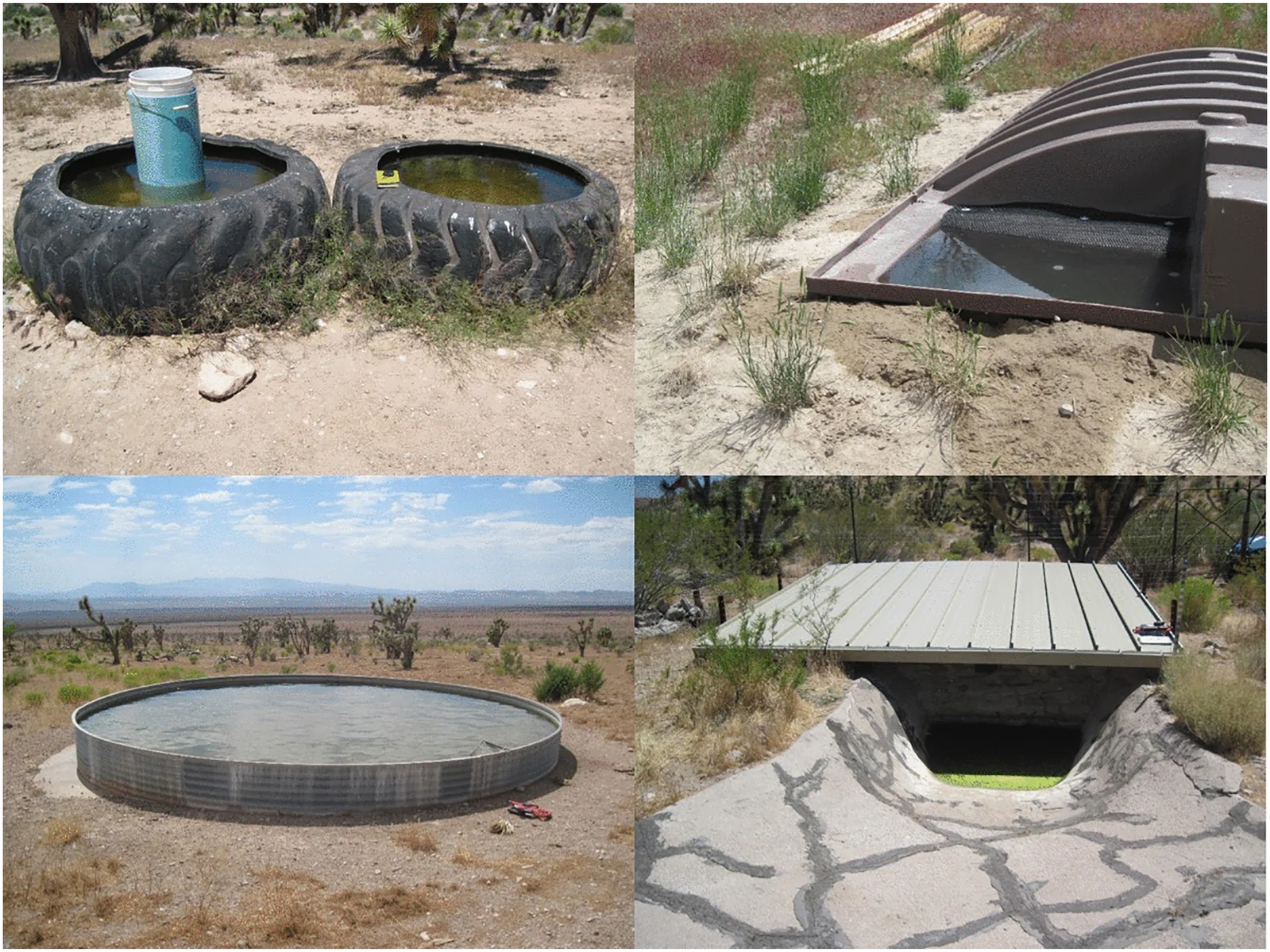
Examples of artificial water sites surveyed to detect Great Basin spadefoot toads (*Spea intermontana*) and red-spotted toads (*Anaxyrus punctatus*). Data were collected in the Great Basin and Mojave Deserts from 2011.

The Mojave Desert study region included 1,064 km^2^ of publicly accessible land in extreme southwestern Utah, northwestern Arizona, and southeastern Nevada managed by the United States Department of the Interior, Bureau of Land Management (Fig 1). The landscape alternated between rolling hills and dry washes that gradually emptied into the Beaver Dam Wash. Common vegetation included creosote (*Larrea tridentata*), Joshua tree (*Yucca brevifolia*), black brush (*Coleogyne ramosissima*), and red brome (*B. rubens*). Along the foothills of the Beaver Dam Mountains, vegetation consisted of sagebrush and juniper, transitioning to pinyon pine (*Pinus edulis*) at higher elevations. Elevations ranged from 819 m to 1526 m. Based on [1981–2010] PRISM climate normals spatially averaged across the study region, the Mojave Desert had a mean annual air temperature of 17.7°C. The annual averages of daily minimum and maximum temperatures were 11.5°C and 23.9°C, respectively, and mean annual precipitation was 195.2 mm [39]. We identified 50 AWS, including wildlife water catchments and livestock drinking troughs, in the Mojave study region using locations provided by the Utah Department of Natural Resources and the Bureau of Land Management as well as exploring all recognizable roads from satellite imagery (Figs 1, 2).

### Data collection

From May to July of 2011, we conducted nocturnal visual encounter surveys for toads at all known AWS in both study regions. Each AWS was visited once at night with two surveyors. The entire edge of the AWS was walked and surveyed visually for toads [40]. Species were determined by visual inspection according to *A Field Guide to  Western reptiles and Amphibians* [41]. Only the presence of adults was recorded; larvae were not sampled. Due to the spatial extents of our study regions, we could not visit each AWS within a single night. Therefore, on each sample night, we surveyed a new, previously unsampled AWS and as many neighboring unsampled sites as possible within the hours of 2100–2300. At each AWS, we recorded the species present to the lowest possible taxonomical level. No permits were required for this research as desert toads were not handled or collected during nighttime surveys. No protected species were sampled in our study. To test our hypotheses, we focused on acquiring variables for design, isolation, terrain, and vegetation for each AWS.

*Design*—These variables were acquired on site and were intended to capture representative variation associated with the design, composition, and dimensions of each AWS. We determined access height of each AWS by measuring the distance (cm) between ground level and the lowest access point to water, as amphibians will often take the least energetically costly route. We assessed presence of overhead infrastructure cover as present or absent. We measured the perimeter around each AWS as the total length (m) of the shoreline/edge. We used perimeter and the shape of the AWS to calculate the surface area (m^2^). Lastly, we determined the composition of each AWS (e.g., metal, concrete). We included a categorical variable ‘target species’ to account for the species for which the AWS was developed and these categories included big game, upland game, livestock, and none.

*Isolation*—We measured several isolation metrics associated with AWS using ArcGIS Pro (Environmental Systems Research Incorporated, Redlands California, USA). We determined distances from AWS to the nearest natural water source, the nearest water source of any type, the nearest occupied water source, and the nearest drainage. We also estimated separate densities of water sources (as another measure of isolation) within 0.5, 1, 3, and 5 km radii around each AWS [see 42].

*Terrain*—We used ArcGIS Pro to characterize and calculate local terrain metrics surrounding each AWS from a 30-m resolution digital elevation model (DEM). These metrics included elevation (standardized by study region), slope, terrain ruggedness, and topographic position index (TPI). We calculated terrain ruggedness within a 90-m radius of each AWS [43]. The TPI metric was calculated for the focal 30-m cell relative to the mean elevation of its surrounding 3 × 3 neighborhood. Higher TPI values indicated high points on the landscape (e.g., ridges) whereas lower TPI values indicated low points (e.g., drainages). We also included the available water capacity (AWC) of the soil and soil texture category at each AWS [44].

*Vegetation*—We included variables for both aquatic and terrestrial vegetation. For aquatic vegetation, we initially attempted to include emergent and floating vegetation as separate binary variables. But emergent vegetation was only observed at 10% (7/68) of sites; therefore, we combined emergent and floating vegetation into a single binary ‘aquatic vegetation’ variable to represent presence (1) or absence (0) of vegetation. As the purpose of our study was to identify potential characteristics of AWS that were associated with toad presence, we included estimates for cover and height around AWS using the Random T-Square method along 30-m transects in all four cardinal directions. The Random T-Square method [45] is used to estimate vegetation density by measuring the distance from a randomly selected point to the nearest plant and the distance from that plant to its neighboring plant. Further, because desert toads may cue in on dense patches of riparian vegetation, characteristic of natural water sources, we also recorded visual obstruction at each AWS using a Robel pole [46]. For these measurements, we placed a Robel pole within 1 m of the edge of AWS and recorded how much of the pole was visible at distances of 5, 10, 15, 20, and 50 m in all four cardinal directions.

To account for other potential factors driving the presence of desert toads, aside from those related to our hypotheses, we included weather covariates extracted from Copernicus Climate Change Service (C3S) climate reanalysis products [47]. Air temperature (2-m air temperature) and wind speed (derived from 10-m wind components) were obtained from the ERA5-Land hourly reanalysis (9-km spatial resolution) [48], whereas total cloud cover was obtained from the ERA5 hourly atmospheric reanalysis (31-km spatial resolution) [49]. Hourly values were extracted for the geographic coordinates and survey hour of each AWS. These metrics included several variations of precipitation as desert toads are known to respond quickly to precipitation [13]: presence of any rain in the 24 h before the survey, total precipitation (mm) in the 24 h before the survey, total precipitation (mm) in the 72 h before the survey, presence of ≥0.2 mm of rain in the 24 h before the survey, and presence of ≥1 mm of rain in the 24 h before the survey. We determined the number of days since last rainy hour (≥0.2 mm), including a second metric capped at seven days to reduce the influence of prolonged dry periods. We also recorded whether rain occurred on the survey day or either of the two preceding days. We included measures of temperature (2-m resolution), wind speed (10-m resolution), and percent cloud cover. We included a binary variable for the ephemeral status of each AWS as the consistent presence of water may affect toad presence. Ephemerality was determined from repeated daytime site visits conducted throughout the study as part of a concurrent project. Sites observed to dry completely at least once during the study period were classified as ephemeral, whereas sites that retained water during all visits were classified as permanent. Lastly, we included a binary variable to indicate the presence of large, domestic ungulates at AWS as the presence of these large species may negatively (e.g., physical exclusion) or positively (e.g., hoof prints to create shallow pools) influence toads.

### Statistical analyses

We modeled toad presence as a binary response (1 = at least one species of toad present, 0 = no species of toad detected) using generalized linear mixed-effects models with a binomial error distribution and logit link in R version 4.5.2 [50] using the `glmmTMB` package version 1.1.14 [51]. We used this pooled species response because our primary objective was to evaluate AWS characteristics associated with use by desert toads generally and because the number of detections did not support conducting the complete model-selection analysis separately for each species. We included survey date as a random intercept to account for potential nonindependence among AWS surveyed on the same night. Because each site was sampled only once, site was not included as a random effect. Region (Great Basin or Mojave) and standardized Julian date were included as fixed effects in every candidate model, including the null model, to account for regional differences in toad species and temporal progression of the breeding season, respectively. Continuous explanatory variables were centered and scaled before analysis; surface area and perimeter were natural-log transformed prior to centering and scaling because of their skewed distributions.

We used an information-theoretic model-selection approach based on the small-sample corrected Akaike information criterion [AICc; 52] to evaluate the presence of toads as a function of AWS design, isolation, terrain, and vegetation characteristics. Given our modest sample size of 68 AWS and the large number of potential explanatory variables, we used a three-stage modeling approach to reduce the risk of overfitting and limit the inclusion of uninformative variables. The null model used throughout the analysis contained the forced effects of region and standardized Julian date and the random intercept for survey date, but no focal environmental or AWS predictor.

In stage one, we evaluated environmental variables that could explain variation in toad presence independently of the AWS characteristics represented by our hypotheses. Each candidate model included region and Julian date as forced fixed effects and survey date as a random intercept, as described above. We then added one environmental variable at a time to this baseline model, as well as four models containing biologically plausible two-way interactions. Each interaction model included the occurrence of rain within the preceding 24 h, the second environmental predictor, and their interaction term. We compared these models using AICc. Environmental variables were advanced to subsequent stages only when models containing them received greater support than or were not competing with the null model.

In stage two, we constructed a separate model for each explanatory variable within the four AWS categories: design, isolation, terrain, and vegetation. Each candidate model included region and Julian date as forced fixed effects and survey date as a random effect, along with one focal explanatory variable. Models were compared separately within each AWS category, and models within 2 ΔAICc units of the top-ranked model were considered competitive [52]. Variables from competitive models were advanced to stage three only when their models received greater support than the null model. Because models in stages one and two contained only one focal explanatory variable, we did not exclude variables solely based on correlations among focal predictors before fitting these models. Thus, correlated predictors could be evaluated as alternative representations of related ecological processes without occurring together in the same model. Before constructing the stage-three composite models, we calculated pairwise correlations among all variables that advanced from stage two. Predictors with ∣r∣ > 0.3 were considered correlated and were not included together in the same composite model. When correlated predictors represented the same conceptual category, we retained the predictor associated with the lower AICc value in stage two [53].

In stage three, we constructed composite models containing different combinations of the variables advanced from stage two. These models allowed us to compare the best-supported predictors across AWS categories within a common modeling framework. We compared the composite models using AICc and calculated ΔAICc values and Akaike model weights. Following model selection, we calculated coefficient estimates, associated standard errors, and 95% confidence intervals for the top-supported model. We then plotted the predicted probability of toad presence as a function of the supported explanatory variables.

Model adequacy was evaluated using 1,000 simulation-based quantile residual replicates generated with the `DHARMa` package version 0.4.7 [54] and confirming the absence of convergence warnings. We visually inspected residuals against fitted values and continuous explanatory variables and tested for departures from residual uniformity, overdispersion or underdispersion, and excess outliers. We also visually examined a Q–Q plot of the survey-date random effects for substantial departures from the assumed normal distribution.

## Results

In the Great Basin, we detected Great Basin spadefoot toads (*Spea intermontana*) at 61% (11/18) of the sampled AWS. In the Mojave, we detected red-spotted toads (*Anaxyrus punctatus*) at 38% (19/50) of the sampled AWS. Additionally, we observed two other toad species in the Mojave: the Arizona toad (*A. microscaphus*) at 2% (1/50) and Woodhouse’s toad (*A. woodhousii*) at 2% (1/50) of sampled AWS. Both additional detections occurred at AWS where red-spotted toads were also detected; therefore, including these species in the pooled presence response did not change the presence/absence classification of any site.

None of the models in stage one of analysis significantly outperformed the ‘null’ model (Table 1). Two models had lower AICc scores than the ‘null’ model but were within 0.66 AICc points of the ‘null’. None of these variables improved model fit or outperformed the ‘null’ model, so they were not advanced to the next stage.

**Table 1:** Stage one model selection results for the presence of Great Basin spadefoot toads (*Spea intermontana*) and red-spotted toads (*Anaxyrus punctatus*) as a function of environmental variables. K: number of model parameters; LL: log likelihood of model; AICc: Akaike’s Information Criteria corrected for small samples; Weight: model weight. All candidate models included the same baseline structure: region and standardized Julian date as fixed effects and survey date as a random effect. Terms listed under “Model” represent predictors added to this baseline structure. For interaction models, the model included both constituent main effects and their interaction term (e.g., ‘Rain 24 h × Temperature’ represents ‘Rain 24 h + Temperature + Rain 24 h × Temperature’). Data were collected in the Great Basin and Mojave Deserts from 2011.

Model*	K	LL	AICc	∆ AICc	Weight
Any_precip_24h	5	−41.64	94.25	0.00	0.16
Wind speed	5	−41.83	94.63	0.39	0.13
Null	4	−43.13	94.90	0.66	0.11
Precip (mm) 24h	5	−42.09	95.14	0.89	0.10
Days since rain (7d cap)	5	−42.65	96.27	2.02	0.06
Any precip 24h × Days since rain (7d cap)	7	−40.25	96.37	2.12	0.05
Precip 24h > 1mm	5	−42.72	96.41	2.16	0.05
Rain 3d	5	−42.78	96.53	2.28	0.05
Precip 24h > 0.2mm	5	−42.92	96.80	2.56	0.04
Cloud cover %	5	−43.01	96.99	2.75	0.04
Precip (mm) 72h	5	−43.12	97.20	2.95	0.04
Ephemeral	5	−43.12	97.21	2.96	0.04
Temperature	5	−43.12	97.21	2.96	0.04
Days since rain	5	−43.13	97.22	2.98	0.03
Domestic ungulates	5	−43.13	97.23	2.98	0.03
Any precip 24h × Wind speed	7	−41.19	98.24	3.99	0.02
Any precip 24h × Cloud cover %	7	−42.12	100.10	5.85	0.01
Any precip 24h × Temperature	7	−42.42	100.71	6.46	0.01

In stage two, models from the design category were the only ones that outperformed the null. ‘Access height’ and ‘surface area’ models were the top models from the design category, and these accounted for 78% of the model weight (Table 2). Both ‘access height’ and ‘surface area’ were advanced to stage three. For isolation, terrain, and vegetation categories, the ‘null’ model was either the top model or a competing model (< 2 ΔAICc of the top model) and therefore, no models from these categories were advanced to stage three (Table 2).

**Table 2:** Stage two model selection results for the presence of Great Basin spadefoot toads (*Spea intermontana*) and red-spotted toads (*Anaxyrus punctatus*) across artificial water site categories. K: number of model parameters; LL: log likelihood of model; AICc: Akaike’s Information Criteria corrected for small samples; Weight: model weight. All candidate models included the same baseline structure: region and standardized Julian date as fixed effects and survey date as a random effect. Terms listed under “Model” represent focal AWS predictors added to this baseline structure. Data were collected in the Great Basin and Mojave Deserts from 2011.

Category	Model	K	LL	AICc	∆ AICc	Weight
*Design*	Access height	5	−39.34	89.66	0.00	0.42
	Surface area	5	−39.50	89.97	0.31	0.36
	Perimeter	5	−40.62	92.20	2.54	0.12
	Material surface	5	−41.87	94.71	5.05	0.03
	Null	4	−43.13	94.90	5.24	0.03
	Target species	7	−39.91	95.68	6.02	0.02
	Roof cover	5	−42.61	96.19	6.54	0.02
	Composition	9	−38.25	97.60	7.94	0.01
*Isolation*	Distance to drainage	5	−41.17	93.30	0.00	0.27
	Distance to nearest occupied	5	−41.87	94.71	1.42	0.13
	Null	4	−43.13	94.90	1.60	0.12
	Water density 0.5 km	5	−42.00	94.97	1.68	0.12
	Distance to nearest neighbor	5	−42.01	94.99	1.69	0.12
	Water density 1 km	5	−42.36	95.68	2.39	0.08
	Water density 3 km	5	−42.53	96.04	2.74	0.07
	Water density 5 km	5	−42.92	96.81	3.51	0.05
	Distance to natural	5	−43.10	97.16	3.86	0.04
*Terrain*	Elevation	5	−41.05	93.07	0.00	0.39
	Null	4	−43.13	94.90	1.83	0.16
	TPI 30m	5	−42.04	95.06	1.98	0.15
	Slope	5	−42.34	95.64	2.57	0.11
	Soil texture type	6	−41.29	95.95	2.88	0.09
	Ruggedness	5	−43.08	97.12	4.05	0.05
	Soil water capacity	5	−43.13	97.23	4.16	0.05
*Vegetation*	Null	4	−43.13	94.90	0.00	0.23
	Aquatic vegetation	5	−42.24	95.45	0.54	0.18
	Shrub cover	5	−42.72	96.41	1.51	0.11
	Robel 5m	5	−42.94	96.84	1.94	0.09
	Shrub height	5	−42.95	96.86	1.96	0.09
	Robel 15m	5	−42.98	96.93	2.03	0.08
	Robel 20m	5	−42.98	96.93	2.03	0.08
	Robel 50m	5	−43.09	97.15	2.25	0.07
	Robel 10m	5	−43.13	97.23	2.32	0.07

Stage three was the second of two rounds of model selection evaluating the relative importance of different AWS characteristics on toad presence. The only AWS category represented in this final round of model selection was AWS design (Table 3). We found a single top model in the design category ‘access height + surface area’, which accounted for 86% of the model weight (Table 3). We calculated model coefficients for this top model, and of these coefficients, ‘access height’ and ‘surface area’ had confidence intervals not encompassing zero (Table 4). ‘Access height’ was negatively associated with the presence of toads whereas ‘surface area’ revealed a positive association (Fig 3). All models retained for inference converged successfully. Simulation-based residual diagnostics for the final model indicated no significant evidence of residual nonuniformity (Kolmogorov–Smirnov test: P = 0.525), overdispersion or underdispersion (P = 0.600), or excess outliers (P = 1.000). The combined adjusted quantile test also detected no significant residual pattern across predicted values.

**Table 3:** Stage three model selection results for the presence of Great Basin spadefoot toads (*Spea intermontana*) and red-spotted toads (*Anaxyrus punctatus*) at artificial water sites. K: number of model parameters; LL: log likelihood of model; AICc: Akaike’s Information Criteria corrected for small samples; Weight: model weight. All candidate models included the same baseline structure: region and standardized Julian date as fixed effects and survey date as a random effect. Terms listed under “Model” represent focal AWS predictors added to this baseline structure. Data were collected in the Great Basin and Mojave Deserts from 2011.

Model	K	LL	AICc	∆ AICc	Weight
Access height + surface area	6	−35.64	84.66	0.00	0.86
Access height	5	−39.34	89.66	5.00	0.07
Surface area	5	−39.50	89.97	5.31	0.06
Null	4	−43.13	94.90	10.24	0.01

**Table 4:** Top model estimates for the presence of Great Basin spadefoot toads (*Spea intermontana*) and red-spotted toads (*Anaxyrus punctatus*) at artificial water sites. SE: standard error of the mean; LCL: lower 95% confidence level; UCL: upper 95% confidence level; *z*: z-value; *p*: p-value; OR (CI): odds ratio and confidence interval. Continuous predictors were centered and standardized before analysis; therefore, odds ratios represent the change in odds of toad presence for a one-standard-deviation increase in each predictor. Surface area was natural-log transformed before standardization. Model included fixed effects for region and standardized Julian date and a random effect for survey date. Data were collected in the Great Basin and Mojave Deserts from 2011.

Coefficient	Estimate	SE	*z*	*p*	LCL	UCL	OR (CI)
Intercept	0.29	0.86	−1.40	1.98	0.34	0.74	1.34 (0.25–7.27)
Access height	−1.06	0.46	−1.96	−0.15	−2.29	0.02	0.35 (0.14–0.86)
Surface area	1.31	0.64	0.05	2.58	2.04	0.04	3.72 (1.05–13.16)
Region	−0.77	0.97	−2.67	1.14	−0.79	0.43	0.46 (0.07–3.12)
Julian date	−0.39	0.44	−1.24	0.46	−0.90	0.37	0.68 (0.29–1.59)

**Fig 3:**
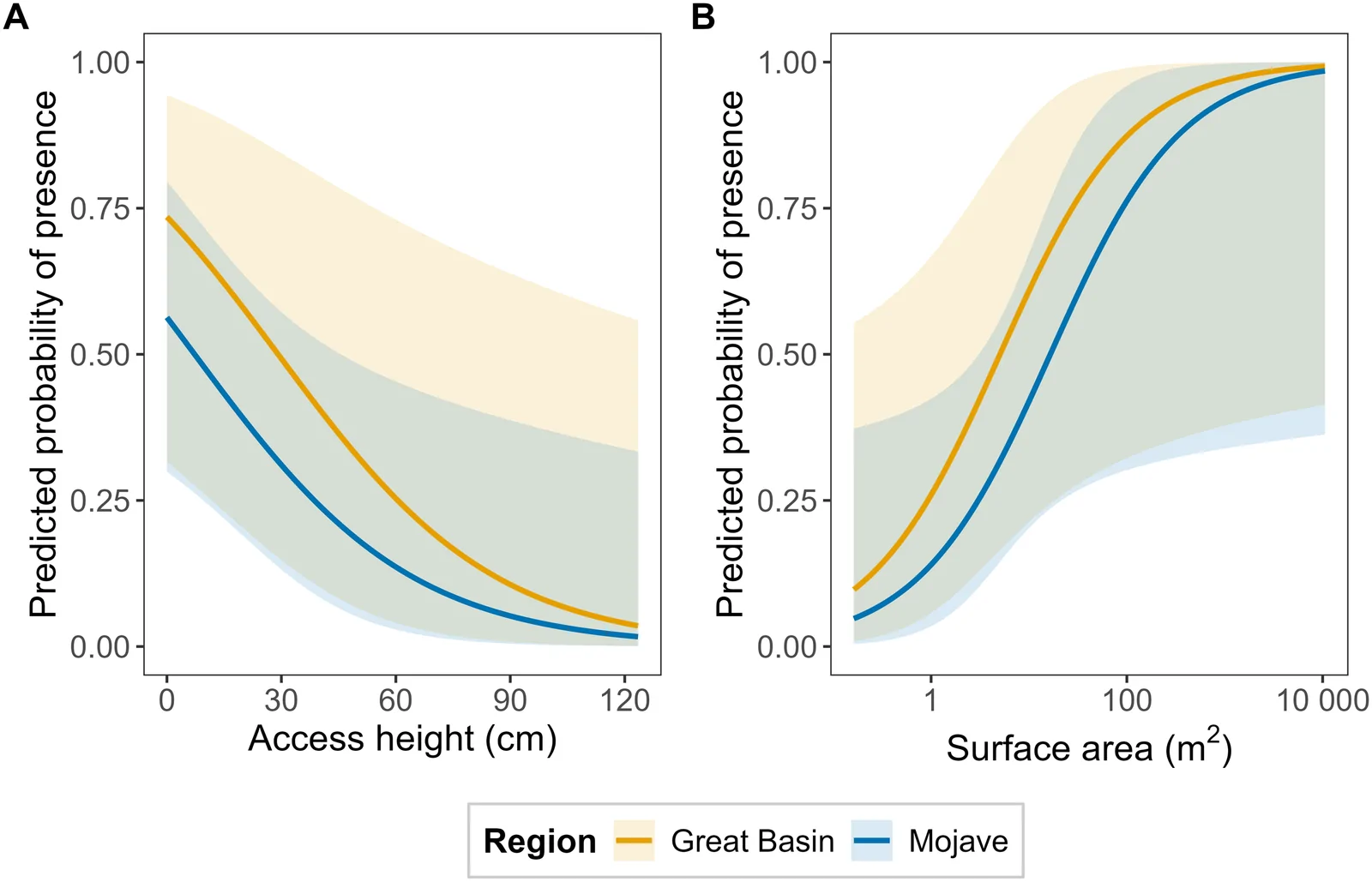
Model predictions for Great Basin spadefoot toads (*Spea intermontana*) and red-spotted toads (*Anaxyrus punctatus*) presence as a function of (A) access height to water and (B) surface area at artificial water sites. The shaded error lines represent the 95% confidence intervals. Data were collected in the Great Basin and Mojave Deserts from 2011.

## Discussion

We found that desert toads at a minor yet appreciable proportion of the AWS in both desert systems. The design hypothesis (i.e., the likelihood of toad presence increases with AWS characteristics that facilitate access to water) was the only supported hypothesis in our study. Of all the variables tested in this study, only ‘access height’ and ‘surface area’ consistently explained the presence of toads at AWS. Landscape positioning and local environmental factors were not significant contributors to toad presence at AWS.

Access height of AWS was one of the most informative variables in this study. Because most AWS are designed for livestock or game species, these features are not always suitable for non-target species, such as desert toads. Among the AWS surveyed in our study, the average access height was 20.8 cm, which is likely beyond the vertical jump of a desert toad. That said, desert toads are likely better climbers than jumpers. For example, Hudson, Brown [55] found that 60% of cane toads (*Rhinella marina*) were able to climb out of a vertical mesh cylinder 50 cm tall. However, several AWS in our study were possibly too tall or difficult to climb (e.g., corrugations in metal) for desert toads. Our findings corroborate similar observations from other research showing that Natterjack toads (*Epidalea calamita*) and European green toads (*Bufotes viridis*) were unable to cross vertical concrete barriers of 13–24 cm in height and additions of overhangs on the concrete barrier reduced climbing even further [31]. Great Basin spadefoot and red-spotted toads, like most desert toads, are small-bodied and the high access points at some AWS in our study may physically prevent these species from accessing water or increase the energetic cost of climbing. Making AWS access points flush with ground or with gently sloping access improves accessibility for desert toads [see 22, 56].

While there is limited published information about the effect of AWS surface area on toad presence, studies of natural water sources provide some relevant context. Previous research has found that the area of breeding habitat (i.e., surface area) at natural water sources was positively associated with anuran presence including red-spotted toads and northern leopard frogs (*Rana pipiens*) [57,58]. We found a similar positive association between AWS surface area and toad presence. Larger AWS may provide a larger target for dispersing toads and therefore increase the likelihood that individuals encounter a water source while navigating the landscape. Larger water surfaces may also provide more available breeding or aquatic habitat. Although we did not evaluate the mechanisms underlying this relationship, our findings are consistent with positive associations between water source size and anuran presence reported at natural water sources in desert environments.

Our understanding of the vagility of desert toads is not clear and this lack of knowledge impedes our understanding of how AWS fit into their spatial ecology. Understanding the vagility of desert toads is challenging due to the limited number of studies covering this topic; existing research reports variable distances ranging from ~10 m up to 800 m [9,33,34]. Most distances traveled are relatively short, with fewer long-distance movements. While we did not record distances traveled by toads, we observed toads at sites exceeding 800 m from the nearest occupied artificial or natural water source (Great Basin: mean = 1504 m, min = 19 m, max: 5745 m, n = 11; Mojave: mean = 1977 m, min = 14 m, max: 4192 m, n = 19). Our findings reinforce the idea that desert toads may disperse farther than what has been recorded [57]. However, these apparent long-distance movements should be interpreted with caution, as our isolation metrics were based on mapped, known water sources; unreported or ephemeral water features (e.g., shallow depressions, clay pans) that form temporarily after precipitation may have gone undetected, potentially facilitating dispersal between AWS and biasing our distance estimates. This may partly explain why, based on what little has been published along with our findings, spatial isolation does not appear to be a driver of toad presence at desert water sources [57].

Local vegetation and terrain characteristics at AWS did not influence the likelihood of desert toad presence. While aquatic vegetation has been shown to be an important habitat factor for amphibians occupying AWS and natural water sources [57,59], our findings did not reveal informative relationships for these habitat categories. Elevation was an important predictor for red-spotted toads at natural water sources in the Mojave Desert [57], but elevation was not a significant predictor in our study. While it is difficult to pinpoint these discrepancies without further study, the habitat in or around AWS may be of overall less importance for toad presence than the actual water these features represent [60].

Breeding activity in desert toads is often associated with precipitation events, and some species even exhibit eruptive breeding following rainfall [61], yet weather-related variables did not influence the likelihood of desert toad presence at AWS. While we cannot definitively explain why weather-related variables were not more explanatory, there are several possible explanations. First, precipitation may primarily influence dispersal and reproductive activity rather than sustained occurrence; once toads are established at a site, our response variable (presence/absence) may not jave been sensitive to short term rainfall, as toads may remain at AWS regardless of recent precipitation. Second, ERA5 climate data may have been too coarse spatially and temporally to reflect the microclimatic conditions that desert toads select. Third, desert toads are opportunistic breeders [62] and may select breeding sites based on characteristics of the water source in addition to short-term weather conditions. In temporary water sources, hydroperiod can strongly influence reproductive success because larvae must complete development before the water source dries, and drying conditions can accelerate metamorphic development in some amphibians (Denver et al. 1998). Temperature may also influence larval growth and development (Schiesari 2006). However, most AWS in our study provided relatively consistent water, potentially reducing the importance of brief hydroperiods or recent precipitation in determining use of these sites. Thus, characteristics of the water source itself may have been more important than short-term weather conditions in explaining toad presence at AWS. Fourth, our response variable was presence/absence (not activity or calling intensity), so responses to fine-scale changes in weather may have been difficult to detect. Although we did not detect a weather effect, that does not minimize the ecological importance of these variables from other studies, these simply were not detectable at this scale of analysis.

While our study revealed two potentially important variables that may contribute to the ongoing management and design of AWS in western deserts, our study has potential limitations. First, our findings are constrained to a subset of the AWS present in two western deserts, and as such, may not be generally applicable to other regions or species. Second, as we captured only a single season view of toad presence, this might miss interannual variability among desert toad populations given the sporadic nature of their life history strategies [61]. Third, we did not assess the availability of burrows [6], which may help explain toad presence at AWS due to their role as potential refugia from environmental extremes present in deserts. Fourth, our survey method did not account for imperfect detectability; toads present at an AWS may go undetected during a single nocturnal survey due to their cryptic behavior, brief periods of activity, or partial concealment in vegetation burrows. This could lead to underestimating actual use of AWS by desert toads, causing us to misclassify occupied sites as unoccupied and potentially limiting our ability to detect relationships with other covariates. Finally, pooling detections into a single toad-presence response may have obscured species-specific relationships with AWS characteristics. Great Basin spadefoot toads and red-spotted toads differ in aspects of their ecology, and because focal species identity was confounded with study region, we could not separate species-specific responses from regional differences. Consequently, our results describe pooled patterns across the focal species and study regions and should not be interpreted as evidence of identical responses among desert-toad species. Despite these limitations, our study is the first to evaluate relationships between AWS design, local habitat features, and landscape position characteristics with desert toad presence. The statistical strength of ‘access height’ and ‘surface area’ as factors is compelling and warrants additional testing in other deserts and species to determine the robustness of the pattern we observed.

Providing easier access to AWS for desert amphibians may result in some of these waters becoming ecological traps for amphibians [56] and other wildlife. For example, increasing access for toads may increase the risk of drowning for other wildlife species if appropriate precautions and guidance are not followed. Additionally, water quality/chemistry concerns for some AWS have been raised [27,59,60,63]. The presence of AWS may inadvertently attract amphibians and other wildlife to low quality sources of water which negatively affect the survival or reproductive efforts of wildlife. The installation of AWS may also unintentionally attract invasive species [25], potentially counteracting the positive effect on native biodiversity.

Artificial water sites in both the Great Basin and Mojave Deserts are utilized habitat features for desert toads. Amphibians are often overlooked in AWS management [22] as the focus is usually on livestock or game species (i.e., ungulates, birds). Our study highlights the importance of access height and surface area as key modifiable features influencing desert toad use. Landscape position did not play a role in toad presence at AWS. Design modifications, such as lowering access height, avoiding vertical barriers, and where feasible, providing greater accessible surface area, may represent relatively simple management practices to improve the suitability of AWS for desert toads.

## Supporting information

S1 Data Dataset. (CSV)
